# Should We Vaccinate Healthcare Workers Against Respiratory Syncytial Virus?

**DOI:** 10.1111/irv.13363

**Published:** 2024-08-12

**Authors:** Klinger Soares Faico‐Filho, Ana Helena Sita Perosa, Nancy Bellei

**Affiliations:** ^1^ Divisão de Doenças Infecciosas, Departamento de Medicina, Laboratório de Virologia, Escola Paulista de Medicina (EPM) Universidade Federal de São Paulo (UNIFESP) São Paulo Brazil

**Keywords:** health care workers, respiratory syncytial virus, vaccine

## Author Contributions

**Klinger Soares Faico‐Filho:** conceptualization, investigation, writing – original draft, writing – review and editing. **Ana Helena Sita Perosa:** conceptualization, investigation, writing – original draft, writing – review and editing. **Nancy Bellei:** conceptualization, investigation, writing – original draft, writing – review and editing.

## Conflicts of Interest

The authors declare no conflicts of interest.

Dear Editor,

The recent approval of the respiratory syncytial virus (RSV) vaccine for elderly individuals and pregnant women marks a significant milestone in the prevention of this respiratory infection. RSV is known for causing severe respiratory illness, particularly in vulnerable populations. This development highlights the necessity of considering vaccination programs for other high‐risk groups, such as healthcare workers (HCWs), who are frequently exposed to infected individuals and can serve as vectors for nosocomial transmission [1].

Respiratory infections, particularly those caused by RSV, present significant challenges to healthcare systems worldwide, especially in the context of nosocomial transmission. HCWs, due to their frequent exposure to infected individuals, are at increased risk of RSV infection. However, data on the epidemiology of RSV infections among HCWs are limited. Understanding the extent of RSV infection in this group is crucial for implementing effective preventive measures and safeguarding both HCWs and the patients they care.

We performed a retrospective study to investigate RSV infections in nasopharyngeal swabs collected between January 2021 and April 2024 from HCWs with acute respiratory infection (ARI). Nasopharyngeal swabs were collected and placed in 2 mL of sterile lactate Ringer's solution, and RNA was purified using Extracta Kit Fast ‐ DNA e RNA Viral (Loccus, Brazil), according to the manufacturer's instructions. RSV detection was performed by a one‐step real‐time RT‐PCR with oligonucleotides targeting a conserved region of the matrix gene [2] using AgPath‐ID One‐Step RT‐PCR Reagents (Applied Biosystems, USA) with 5 μL of purified RNA, 800 nM of each primer, and 200 nM of the TaqMan probe. The reactions were performed on a Quantstudio 6 Pro Real‐Time PCR System (Applied Biosystems) for 10 min at 50°C and 10 min at 95°C, followed by 45 cycles of 15 s at 95°C, and 30 s at 55°C (data collection). Samples with Ct ≤ 40 were considered positive. Further, RSV subtypes were identified by another real‐time PCR specific for RSV A and B [3] with the same conditions of the screening reaction.

A total of 4367 HCWs aged from 16 to 92 years (mean 38 ± 13, median 36) was tested, and RSV was detected in 2.6% (115/4367) of HCWs. The highest annual positivity rate was 3.6% in 2022 and the lowest was 1.9% in 2023 (Table 1).

**TABLE 1:** The distribution of RSV positive cases among healthcare workers (HCWs) across different age groups.

	RSV positive	RSV subtypes	
	*n* (%)	RSV A	RSV B
2021 (*N* = 1707)	39 (2.3%)	26 (66.7%)	13 (33.3%)
2022 (*N* = 1260)	46 (3.6%)	6 (13%)	39 (84.8%)
2023 (*N* = 1006)	19 (1.9%)	9 (47.4%)	9 (47.4%)
2024 (*N* = 394)	11 (2.8%)	2 (18.2%)	9 (81.8%)
Age (years)	19–79	19–66	20–79
Mean (DP)	39.2 (13.4)	37.5 (11.7)	39.8 (14.1)
Median	38	35	38.5
≤ 20 years (*N* = 164)	3 (1.8%)	1 (33.3%)	2 (66.7%)
21–30 years (*N* = 1501)	36 (2.4%)	18 (50%)	18 (50%)
31–40 years (*N* = 921)	24 (2.6%)	8 (33.3%)	16 (66.7%)
41–50 years (*N* = 882)	31 (3.5%)	11 (35.5%)	19 (61.3%)
51–60 years (*N* = 670)	12 (1.8%)	1 (8.3%)	11 (91.7%)
> 60 years (*N* = 229)	9 (3.9%)	4 (44.4%)	4 (44.4%)

*Note:* The comparison of proportions was assessed using the chi‐square test for independence. The results indicate no statistically significant difference in the proportions of RSV positive cases among the different age groups (*p* = 0.24).

**FIGURE 1:**
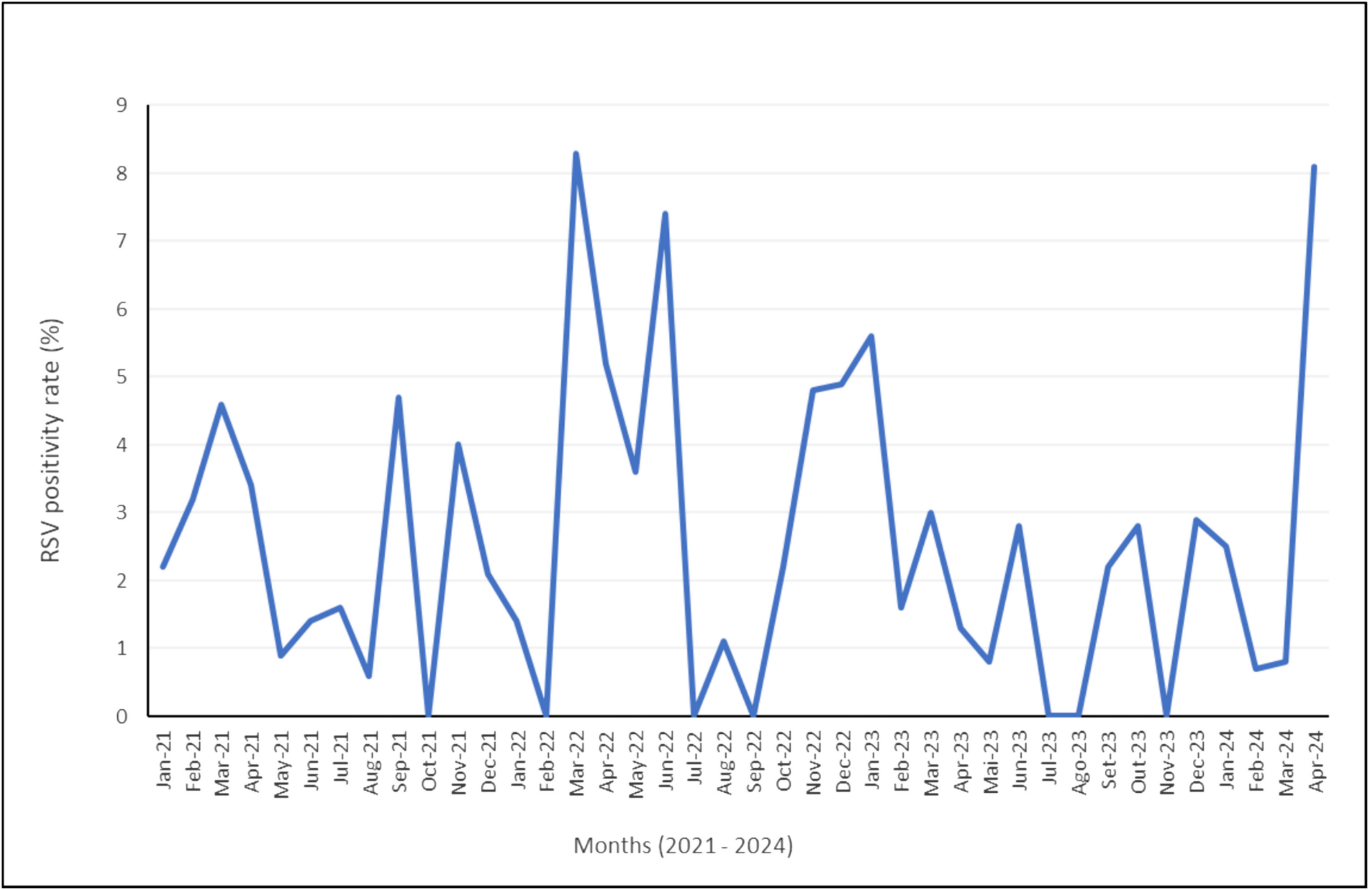
RSV positivity rate in healthcare workers (HCWs) by month, from 2021 to 2024. The line graph depicts the monthly positivity rate of respiratory syncytial virus (RSV) among HCWs from January 2021 to April 2024. The y‐axis represents the RSV positivity rate in percentage, while the x‐axis represents the months and years within the study period. Notable peaks are observed in March 2022 and April 2024, indicating higher RSV positivity rates during these months, with a maximum rate of 8.3% in March 2022. The data highlight fluctuations in RSV infection rates among HCWs over the examined period.

The monthly RSV positivity ranged from 0% to 8.3% (March 2022–April 2024; Figure 1). Regarding RSV subtypes, 37.4% (43/115) were RSV A, 60.9% (70/115) were RSV B, and 1.7% (2/115) were unsubtyped. HCWs over 60 years old showed the highest detection rate (3.9%). Autumn–winter seasons (March–June in our region) showed the highest detections.

HCWs infected with RSV may serve as vectors for transmission within healthcare settings, potentially leading to outbreaks and absenteeism, compromising patient care. The detection of RSV in 8.3% of samples emphasizes the need for infection control measures, preventive strategies in healthcare settings, and implementing surveillance programs to monitor RSV circulation and detect outbreaks early [4].

Given the significant exposure risk and potential for nosocomial transmission, it is essential to consider implementing RSV vaccination programs for HCWs. Protecting these frontline workers may not only safeguard their health but also enhance patient care and reduce the burden of RSV outbreaks in healthcare settings.

## Data Availability

Derived data supporting the findings of this study are available from the corresponding author KSFF on request.
